# Torsional Power and Tip Shape Greatly Change Irrigation Flow Feeding Rate

**DOI:** 10.7759/cureus.33295

**Published:** 2023-01-03

**Authors:** Santaro Noguchi, Asuka Noguchi, Shunsuke Nakakura, Hitoshi Tabuchi

**Affiliations:** 1 Ophthalmology, Saneikai Tsukazaki Hospital, Himeji, JPN; 2 Ophthalmology, Hiroshima University, Hiroshima, JPN

**Keywords:** centurion, torsional phaco, mini-tip, balanced tip, cataract

## Abstract

Background: It is important to maintain intraocular pressure during cataract surgery. When the intraocular pressure sharply drops during phacoemulsification, it is important to ensure a compensatory maximum fluid supply. To the best of our knowledge, this is the first study presenting the maximum irrigation flow supply using an ultrasonic tip shape and torsional power setting.

Methods: The weight of BSS PLUS 500 ejected during torsional oscillation fitted with a mini-tip or balanced tip and nano sleeve with IOP set at 20 mmHg (IOP20) or 40 mmHg (IOP40) was measured. The weight of the BSS ejected from the sleeve over 3.0 s (15 measurements taken at 200-ms intervals) was measured to calculate the irrigation flow feeding rate. Measurements were made four times at each torsional power setting (TP).

Results: With a balanced tip, the irrigation flow rate rose as TP was increased, whereas, at 60% or 90% TP, the irrigation flow rate markedly decreased. With the mini-tip, the irrigation flow rate remained relatively stable, up to 60% or 80% TP but decreased dramatically at higher power settings. Compared with IOP20, the irrigation flow rate increased by 1.21- to 1.28-fold with the balanced tip and by 1.28- to 1.41-fold with the mini-tip at IOP40. At IOP20, the irrigation flow rate was higher with the mini-tip at 0% and 5% TP but equal to or higher with the balanced tip at TP of ≥10%. At IOP40, the irrigation flow rate with the mini-tip was equal to or higher than that with the balanced tip at all TP.

Conclusions: The irrigation flow rate tends to vary with changes in TP and tip shape.

## Introduction

The most important factor for successful phacoemulsification without complications is the maintenance of the anterior chamber [1] via the physiological control of intraocular pressure (IOP) under safe and stable conditions. However, IOP fluctuates widely during cataract surgery [2].

Cataract surgery under high IOP allows the anterior chamber to deepen, increasing the distance between the posterior capsule and ultrasound tip. However, this may cause ocular pain, decreased ocular blood flow, optic nerve fiber damage associated with glaucoma, and corneal edema after cataract surgery [3,4]. Conversely, cataract surgery performed under low IOP causes the anterior chamber to collapse, thereby increasing the risk of aspiration and damage to anterior chamber structures and tissues, including the anterior and posterior capsule, corneal endothelium, and iris [5]. Therefore, maintaining IOP consistently within an appropriate range allows safe cataract surgery.

In passive or gravity-based irrigation systems, IOP cannot be maintained at a constant level depending on the aspiration flow. Although IOP decreases with an increase in aspiration flow rate, phacoemulsification systems with active fluidics allow for the addition of pressure to maintain a constant IOP level [6]. However, most studies have compared irrigation pressure levels without ultrasound oscillation [1,2,6,7]. Such systems passively complement the decreased IOP levels. As ultrasonic oscillation occurs during intraocular irrigation, the specific effects of ultrasound waves on the irrigation flow rate remain unclear. Furthermore, there are multiple types of ultrasound tips. We previously reported that the torsional phacoemulsification amplitude greatly varies with tip shape [7,8].

Therefore, this study aimed to compare two types of ultrasound tips (balanced tips and mini-tips), which differ in shape but have similar other characteristics, such as internal and external diameter, length, and material, to clarify the relationship between irrigation flow rate and power settings [20 torsional power (TP) settings ranging from 5% to 100%] using ultrasound oscillation, thereby determining the effects of power settings on irrigation.

## Materials and methods

This experimental study was conducted at Tsukazaki Hospital, Japan. Phacoemulsification was performed using CENTURION® Vision System (Alcon Laboratories, Inc., Fort Worth, TX, USA) and two ultrasound tips (a semicircular arc-shaped balance tip and bent-shaped mini-tip, a bent tip (0.9 mm Mini ABS 45° Kelman; mini-tip) and semicircular tip (0.9 mm balanced ABS 45° Alcon; balanced tip). A hole with a diameter of 10 mm was drilled into an experimental transparent acrylic cylindrical container (80 mm × 110 mm). The ultrasound tip was inserted and fixed into the experimental container without any contact. The container was covered with an acrylic lid, and the only part accessible to the external world during measurements was a small gap where the handpiece was inserted.

**Figure 1:**
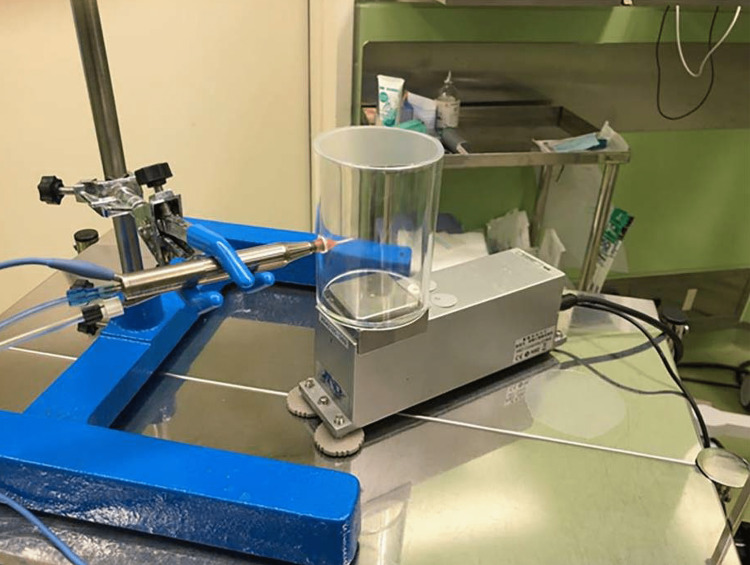
Experimental setting

The container was placed on a digital scale (AD-4212C-300) to obtain its weight in real time (Figure 1). As the purpose of this study was to measure the weight of BSS PLUS 500 intraocular irrigating solution (0.0184%, Alcon) ejected during ultrasound oscillation, measurements were obtained 15 times at 200-ms intervals starting from 3 s after initiating the oscillations. AD-4212C-300 can be used to weigh objects between the maximum and minimum weight limits of 320.084 and 0.001 g, respectively [9].

The ultrasonic tip was inserted and fixed into a cylindrical acrylic container (80 mm × 110 mm) through a 10-mm hole. The container was placed on a digital scale (AD-4212C-300) to obtain its weight in real-time.

A regression line of the change in the weight of BSS solution ejected from the sleeve over 15 measurements was constructed using Microsoft Excel (Redmond, USA), and the slope of this line was defined as the irrigation flow feeding rate. Measurements at each TP were obtained four times, and the mean and standard deviations of the irrigation flow feeding rate were calculated. The experiment was conducted under 20 different TP settings ranging from 0% to 100% in a continuous mode with 5% increments.

The weight of ejected BSS solution during ultrasonic oscillations was measured using a bent tip (0.9 mm Mini ABS 45° Kelman; mini-tip) or semicircular tip (0.9 mm balanced ABS 45° Alcon; balanced tip) and nano sleeve (Alcon) attached perpendicularly to the tip bevel (positioned on each side of the tip), which was placed 0.5 mm at the end of the tip. The CENTURION Vision System (Geneva, Switzerland) was fitted with the handpiece, and the irrigation flow rate was measured at 20 (IOP20) and 40 (IOP40) mmHg at a patient eye level of 0 cm, aspiration flow rate of 20 ml/min, and aspiration pressure of 200 mmHg. The aspiration tube was clamped.

The irrigation flow rate at different ultrasonic power settings was compared with that at 0% TP using the same tip. Next, the irrigation flow rates at two different IOP levels were compared. Finally, the irrigation flow rates were compared based on different tips.

Mann-Whitney U test was used to analyze statistical differences between various measurements. For exploratory analysis, multiple testing corrections were not performed. A p-value of <0.05 was considered to indicate statistical significance. JMP14 (version 14.3; SAS, Inc., Cary, NC) was used for statistical analysis.

## Results

Comparison with 0% TP

Under the condition of using a balanced tip and nano sleeve at IOP20 (BN20 condition), the mean flow rates at 20%, 25%, and 30% TP were significantly higher than those at 0% TP (all p < 0.05, Mann-Whitney U test). Conversely, the mean flow rates at 90%, 95%, and 100% TP were significantly lower than those at 0% TP (p < 0.05). Furthermore, the mean flow rate decreased with an increase in TP from 90% to 100%.

**Figure 2:**
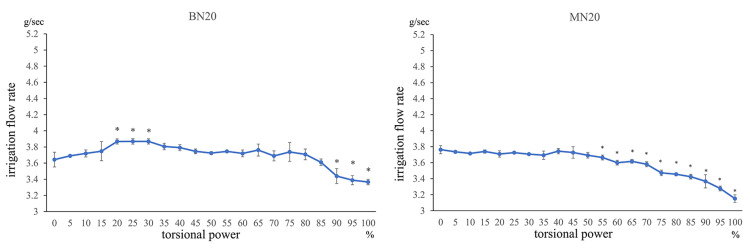
Comparison between 0 torsional power and all torsional powers at IOP 20 mmHg

An increase in flow rate at 20%, 25%, and 30% TP, which was observed under the BN20 condition, was not observed under the condition of using a mini-tip and nano sleeve at IOP20 (MN20 condition), and the irrigation flow rate significantly decreased with an increase in TP from 55% to 100% (all p < 0.05; Figure 2).

Comparison of torsional power and irrigation flow rate under the condition of using a balanced tip and nano sleeve at IOP20 (BN20) or mini-tip and nano sleeve at IOP20 (MN20) (Mann-Whitney U test, *p < 0.05; irrigation flow rate compared with torsional power setting at 0%)

Compared with 0% TP, the irrigation flow rate increased at 5% TP under BN20 condition but began to decrease with a further increase in TP at ~85% (p < 0.05). The irrigation flow rate did not increase under MN20 conditions but significantly decreased with an increase in TP from 55% to1 00% (all p < 0.05).

**Figure 3:**
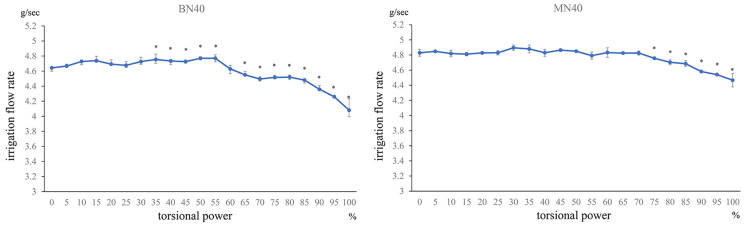
Comparison between 0 torsional power and all torsional powers at IOP40

Under the condition of using the balanced tip and nano sleeve at IOP40 (BN40 condition), the irrigation flow rate at 30%-55% TP was significantly higher than that at 0% TP (all p < 0.05). However, the irrigation flow rate decreased at ≥65% TP (all p < 0.05). Under the condition of using the mini-tip and nano sleeve at IOP40 (MN40 condition), the irrigation flow rate at high-power settings of ≥75% was significantly decreased compared with that at 0% TP, showing decreasing rates with an increase in TP (all p < 0.05; Figure 3).

Comparison of torsional power and flow rate under the condition of using the balanced tip and nano sleeve at IOP40 (BN40) or mini-tip and nano sleeve at IOP40 (MN40) (Mann-Whitney U test, *p < 0.05; irrigation flow rate compared with torsional power setting at 0%). Compared with 0% TP, under BN40 condition, the irrigation flow rate was high up to 60% and increased significantly at 10%, 15%, and 30%-55% TP (all p < 0.05). However, at ≥65% TP, the irrigation flow rate decreased as the power increased (all p < 0.05). Under the MN40 condition, the irrigation flow rate at ≥75% TP significantly decreased compared with that at 0% TP, with rates decreasing with an increase in TP (all p < 0.05).

BN20 vs. MN20

**Figure 4:**
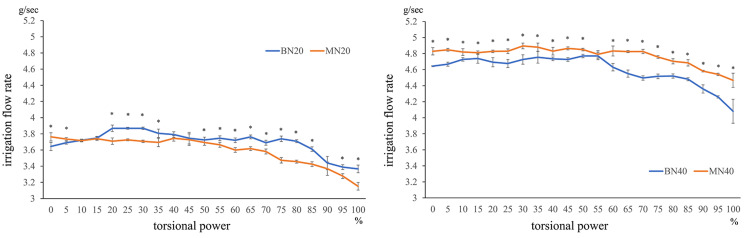
Comparison of irrigation flow feeding amount of mini-tip and balanced tip at IOP20

In a comparison of irrigation flow rates at equal IOP, i.e., between BN20 and MN20 conditions, a significantly higher irrigation flow rate was observed for MN20 condition at 0% and 5% TP (all P < 0.05); however, at ≥20% TP, the flow rate was significantly higher for BN20 condition, except for 40%, 45%, and 90% TP (all p < 0.05). More prominently, the comparison of irrigation flow rates between BN40 and MN40 conditions revealed that the irrigation flow rate measured using the mini-tip was significantly higher for all power settings except 55% TP (all p < 0.05; Figure 4).

For each torsional power setting, the irrigation flow rate measured using the balanced tip and nano sleeve at IOP20 (BN20) was compared with that measured using the mini-tip and nano sleeve at IOP20 (MN20), whereas the irrigation flow rate measured using the balanced tip and nano sleeve at IOP40 (BN40) was compared with that measured using mini-tip and nano sleeve at IOP40 (MN40) (Mann-Whitney U test, *p < 0.05). The irrigation flow rate was higher under the MN20 condition at 0% and 5% TP (all p < 0.05) but was significantly higher under the BN20 condition at 20%-35%, 50%-85%, 95%, and 100% TP (all p < 0.05). The comparison of irrigation flow rates between BN40 and MN40 conditions revealed that the irrigation flow rate measured using the mini-tip was significantly higher for all power settings except 55% TP (all p < 0.05).

IOP20 vs. IOP40

**Figure 5:**
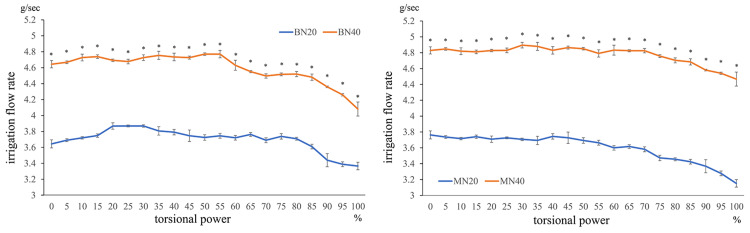
Comparison of irrigation feeding amount of IOP20 and IOP40 for mini-tip or balanced tip, respectively

In a comparison of irrigation flow rates measured using the same tip (balance tip), the irrigation flow rate under BN40 condition was 1.21- to 1.28-fold higher than that under BN20 condition at all power settings (all p < 0.05). When the same comparison was performed using the mini-tip, the irrigation flow rate under MN40 condition was 1.28- to 1.42-fold higher than that under MN20 condition at all TPs (all p < 0.05) (Figure 5).

For each torsional power setting, the irrigation flow rate measured using the balanced tip and nano sleeve at IOP20 (BN20) was compared with that measured using the balanced tip and nano sleeve at IOP40 (BN40), whereas the irrigation flow rate measured using the mini-tip and nano sleeve at IOP20 (MN20) was compared with that measured using mini-tip and nano sleeve at IOP40 (MN40) (Mann-Whitney U test, *p < 0.05). The irrigation flow rate under BN40 conditions was 1.21- to 1.28-fold higher than that under BN20 conditions at all power settings (all p < 0.05). The irrigation flow rate under MN40 conditions was 1.28- to 1.42-fold higher than that under MN20 conditions at all TP (all p < 0.05).

## Discussion

Using the semicircular balanced tip, the irrigation flow rate increased at ~30% TP with IOP20 and ~50% TP with IOP40 but decreased markedly at higher power settings. Using a bent tip, the irrigation flow rate remained unaffected at 50% or 70% TP but notably decreased at higher power settings.

In the comparison between tips, the irrigation flow rate at IOP20 was generally higher using the balanced tip, whereas the irrigation flow rate at IOP40 was higher at all power settings using the mini-tip. At both IOP20 and IOP40, the irrigation flow rate was higher using the mini-tip at 0% TP.

When the irrigation pressure was increased from IOP20 to IOP40, the pressure setting increased by 2-fold but the actual irrigation flow rate increased by only 1.28-fold using the balanced tip and 1.42-fold using the mini-tip, indicating that the irrigation flow rate increases at a higher rate using the mini-tip.

When the tip of the phacoemulsification is completely occluded by a lens fragment during phacoemulsification, the vacuum builds up until a preset vacuum limit is reached. Once the occlusion is cleared (after the lens piece is emulsified/removed), fluid flows from the anterior chamber (positive pressure, small volume) toward the pump/cassette (negative pressure, large volume), resulting in a decrease in IOP, which is known as the surge. Fluctuations in the IOP during surge can lead to shallow anterior chamber or iris/posterior capsule trauma [10].

In conventional phacoemulsification systems, the infusion bottle height or pressurized infusion level is preset to balance irrigation and aspiration and reduce surge effects. Persistent reduction in primary incision size over the past 20 years has led to the requirement of increased injection pressures, resulting in unphysiologically high IOP levels and substantial intraoperative IOP variability [4].

Recently, a new fluidics management system (Centurion Vision System, Alcon Laboratories, USA) was introduced to actively manage irrigation. This system was later updated with an IOP pressure sensor in Phaco Handpiece (Active Sentry Handpiece, Alcon Laboratories, USA) for better performance and faster response to surges. The updated system prevents or mitigates surge events upon the activation of an active sentry [11]. This allows surgeons, for the first time, to perform phacoemulsification at their physiological IOP levels. However, if the IOP level is increased during surgery to prevent capsule rupture due to collapse, the incidence of surge increases [12].

During phacoemulsification, infusion and aspiration are repeated, and the IOP level fluctuates to facilitate the aspiration of the nucleus cataract 3). In particular, when the nuclear fragment passes through the ultrasonic tip, the suction pressure increases, resulting in a surge and IOP collapse 1-3, 7). Recently, a system that actively maintains IOP using ACTIVE FLUIDICS (Alcon) has also been developed. However, both systems compensate by increasing infusion during collapse. This allows the surgeons to achieve recovery from a surge. However, although the supplementary reflux rate may be strongly associated with ultrasonic oscillation, reflux rate, shape of the sleeve and tip, this parameter was not considered in this study.

Until now, no studies have discussed the relationship between the irrigation flow rate, TP, and tip shape. The current study demonstrates that the irrigation flow rate is greatly affected by TP and tip shape. The end of the phaco tip reached maximum amplitude at 80% TP in an S-shaped curve. Above 80% TP, the increase in amplitude becomes negligible and may not contribute to nuclear fragmentation [8]. We speculated that the tip motion induced by torsional ultrasound may be the source of resistance to irrigation fluid ejection and is assumed to decrease consistently with the tip amplitude. However, a comparison with the irrigation flow rate suggested that the change in flow rate was not consistent with the amplitude of the tip end. Although it also depends on the tip type and IOP settings, the irrigation flow rate decreased markedly at high-power settings of ≥80%, wherein the tip point amplitude remains almost constant, suggesting that the resistance to irrigation flow is partially caused by the oscillation of other than the end of the tip. Increasing the TP settings to >80% provides no additional power for nuclear fragmentation and increases the risk of anterior chamber collapse, suggesting that high-power settings are not recommended.

Except for a study reporting the details of a comparison of irrigation systems in cataract surgery devices [6], no previous studies have investigated the relationships between TP and tip type. The ejected quantities were measured under atmospheric pressure in our experiment and are probably similar to the maximal irrigation flow rates at various settings.

Several mechanisms that cause ocular collapse occur during the actual surgery, exemplified by the occlusion break surge and complemented by irrigation flow. However, our results suggest that the irrigation flow required for complementing the anterior chamber greatly varies depending on ultrasonic power, tip, and IOP settings. The semicircular tip has a complex form and is characterized by an increased flow rate at ~30% or 50% TP; however, the irrigation flow rate using the semicircular tip at 0% TP in our study was lower than that using the bent tip at 0% TP. Small amplitude TP (~30% or 50%) oscillations may release the resistance of the water flow to escape between the semicircular tip and sleeve, thereby increasing the flow rate. Conversely, lower TP (0%-50%) oscillations influence the flow rate to a lesser degree using bent tips.

This study revealed that the irrigation flow rate decreased with an increase in TP output using both tips. This suggests that anterior chamber irrigation flow can be insufficient, increasing the risk of anterior chamber collapse in eyes that require high-power TP settings.

If a program automatically increases the IOP level during ultrasonic oscillation when increasing the torsional phaco, the anterior chamber will be more stable. An automatic IOP alteration system with a TP setting would be beneficial for more stable cataract surgeries. Furthermore, the increase in irrigation flow rate also varies between tips; thus, the adoption of irrigation flow rate settings that consider the tip type would also improve the level of safety during surgery.

IOP monitoring systems, such as active fluidics (Alcon) and active sentry (Alcon), respond to changes in pressure that occur passively and do not consider decreases in the flow rate resulting from TP oscillation. Including flow rate settings that consider these variations in advance would allow surgeons to respond more actively to maintain anterior chamber stability.

This experiment did not measure the irrigation flow rate using the device actually inserted in the eye; thus, the influence of insertion through an incision or twist was not considered. Furthermore, because the ultrasonic oscillations occurred in a vacuum, the ejected BSS solution may have volatized and slightly influenced our findings. Finally, the IOP settings were limited to 20 and 40 mmHg, and higher settings may further influence the results.

## Conclusions

In conclusion, the irrigation flow rate varied according to TP and tip shape. The decrease in the irrigation flow rate was particularly significant at higher TP, suggesting that the anterior chamber is more prone to instability in cases that require high-power settings. The relationship among the infusion rate, ultrasonic oscillation, and tip shape, which had not been elucidated until now, was clarified. High-power ultrasonic oscillation causes insufficient intraocular perfusion even if the eyeball is not collapsed. Furthermore, to achieve recovery from the collapsed state, a more pressurized perfusion flow should be supplied during ultrasonic oscillation. The anterior chamber can be stabilized more easily when IOP settings are actively changed during phacoemulsification oscillations according to the tip type and TP.
